# Assessing the Relationship between Prosocial Behavior and Well-Being: Basic Psychological Need as the Mediator

**DOI:** 10.3390/ejihpe13100153

**Published:** 2023-10-07

**Authors:** Linwei Li, Aqeel Khan, Mohd Rustam Mohd Rameli

**Affiliations:** Faculty of Social Sciences and Humanities, Universiti Teknologi Malaysia, 81310 Skudai, Johor, Malaysia; aqeel@utm.my (A.K.); mrustam2@utm.my (M.R.M.R.)

**Keywords:** prosocial behavior, basic psychological needs, well-being, vocational students

## Abstract

Previous research has established a positive link between prosocial behavior (PB) and psychological well-being. However, limited studies have explored the relationship between PB and well-being (WB), particularly among vocational students. Furthermore, the underlying mechanisms, including mediating factors, remain understudied in this context. This study aimed to investigate the association between PB and WB among vocational students while examining the mediating role of basic psychological needs. A sample of 221 vocational students (mean age = 19.68 years, *SD* = 1.57) completed anonymous questionnaires assessing PB, basic psychological needs, and WB. The results revealed a positive correlation between PB and WB in vocational students (*r* = 0.22, *p* < 0.01), with basic psychological needs partially mediating this relationship (*β* = 0.14, *t* = 10.85, *p* < 0.001, 95% *CI* = (0.18, 0.23)). These findings enhance our understanding of the association between PB and vocational students’ WB, shed light on the mechanisms involved, and offer insights into promoting the well-being of vocational students.

## 1. Introduction

Vocational education is critical in preparing students with practical skills for specific careers [1]. Vocational education plays a pivotal role in equipping students with practical skills and knowledge that are directly applicable to specific careers [2]. This form of education is particularly important in the context of China, where the demand for skilled workers is rapidly increasing due to the country’s industrial growth and technological advancements [3]. Vocational education not only provides students with a pathway to gainful employment but also contributes significantly to the overall economic development of the nation [4]. However, research exploring the well-being of vocational students is relatively limited compared to other student populations [5,6]. Understanding the factors contributing to vocational students’ WB is essential for promoting their overall development and educational outcomes. Thus, this study addresses this gap by investigating the association between PB and WB and the mediating role of BPN among vocational students.

Well-being is a complex and multifaceted construct encompassing various dimensions, which include both subjective and objective aspects. In this study, our primary focus centers on a specific facet of well-being among vocational students. This particular facet revolves around individuals’ subjective experiences of happiness, life satisfaction, and vitality, all of which hold significant relevance in educational settings [7]. It has been well-established that higher levels of well-being are associated with numerous benefits for students, for example, improved academic performance, higher levels of engagement, enhanced social relationships, and better mental health [8,9]. To provide clarity regarding the specific dimension of well-being under investigation, it is imperative to underscore our research objectives. In the context of educational outcomes in China, promoting students’ WB has become a crucial objective. Research has demonstrated that students with higher levels of WB tend to exhibit better academic achievements, higher motivation, and improved overall adjustment [10]. Particularly in vocational education, where students are preparing for distinct careers, prosocial behavior holds implications for their future professional interactions and community contributions [11,12]. Consequently, understanding the factors contributing to WB, such as prosocial behavior, is paramount for educational psychologists and practitioners striving to create supportive learning environments and enhance students’ educational experiences. Moreover, this study offers theoretical advancements by proposing and testing a novel framework within the existing literature on prosocial behavior and well-being. This theoretical contribution lays the groundwork for exploring the intricate interplay among prosocial behavior, basic psychological needs, and well-being, thereby enriching the academic discourse on this subject.

Prosocial behavior, characterized by voluntary actions intended to benefit others [13], has been widely recognized as a significant contributor to WB. Engaging in PB fosters positive relationships and social cohesion and contributes to individuals’ WB [14,15]. Furthermore, self-determination theory (SDT) posits that individuals have basic psychological needs for autonomy, competence, and relatedness, which are crucial for optimal psychological functioning and well-being [16]. The fulfillment of these BPNs is positively associated with individuals’ WB [17]. The existing literature examining the relationship between prosocial behavior (PB) and well-being (WB) has presented mixed findings, leaving a research gap in the understanding of the precise nature of this association [18,19,20]. While some studies have reported a positive link between PB and WB, others have yielded conflicting results [21,22]. These discrepancies underscore the complexity of this relationship and highlight the need for more context-specific investigations to better understand the underlying mechanisms at play. Additionally, the mechanisms through which PB influences or affects WB remain unclear and have been understudied [23]. Thus, this study explores the mediating role of BPN in the relationship between PB and WB among vocational students.

## 2. Literature Review

### 2.1. Prosocial Behavior and Well-Being

Prosocial behavior refers to actions that benefit others, groups, or society as a whole [24]. These behaviors encompass acts such as helping, sharing, volunteering, cooperation, and charitable giving [25,26,27,28]. Recent studies have further expanded the understanding of prosocial behaviors, highlighting their relevance in various contexts, including educational settings [29,30]. From an individual standpoint, engaging in prosocial behavior contributes to personal self-esteem and satisfaction [31,32]. On an interpersonal level, prosocial behavior enhances social interactions and fosters interpersonal harmony [33]. Furthermore, prosocial behaviors are considered symbols of social responsibility and play a crucial role in maintaining social harmony [34].

While previous research has primarily focused on contextual factors related to helping behavior, there has been a growing interest in studying prosocial behavior within group and cross-group contexts among students [35]. Specifically, Chinese society places a strong emphasis on interconnectedness and social harmony, valuing virtues such as benevolence, compassion, and mutual support [36]. Acts of helping others are often perceived as integral to nurturing collective well-being and maintaining societal balance [37]. Moreover, the influence of Confucian principles, deeply ingrained in Chinese cultural values, underscores the significance of altruistic actions and moral integrity in interpersonal interactions [38,39]. Within this context, understanding the manifestation and effects of prosocial behavior among students can provide valuable insights into the cultural dynamics and social norms that influence their altruistic actions. Research examining prosocial behavior among students in China can illuminate the cultural factors that shape their attitudes and behaviors, as well as inform the development of culturally sensitive interventions and educational strategies that promote prosocial behavior and enhance the well-being of Chinese students.

With the increasing prominence of psychological issues among vocational students, improving their well-being has become a crucial area of interest for researchers and society at large. Previous research has diligently explored various dimensions of well-being, employing a range of measures to capture the multifaceted nature of this construct across diverse contexts. In this study, we narrow our focus to a specific facet of well-being relevant to vocational students. This facet pertains to a subjective construct, which involves an individual’s comprehensive evaluation of their life based on personal criteria [40]. It encompasses subjective experiences of happiness, life satisfaction, and vitality. Prosocial behaviors, which benefit others and promote harmonious interpersonal relationships, have been linked to well-being [41]. Engaging in prosocial behaviors provides individuals with a sense of purpose and fulfillment [28], promotes positive feelings and moods [42,43], and enhances social integration and connection, leading to the development of interpersonal relationships [44,45,46]. These factors contribute to an improved sense of self-worth, belonging, and connectedness, which ultimately enhances well-being [47,48,49,50].

Based on the aforementioned literature, we propose the following hypothesis:

**Hypothesis** **1.** *There is a positive association between prosocial behaviors (PB) and well-being (WB). In other words, individuals who engage in more prosocial behaviors will have higher levels of well-being.*

However, the underlying mediating mechanisms (i.e., how PB influences WB) among vocational students remain a topic for ongoing inquiry, even though PB has been found to increase levels of WB. Additionally, previous research has explored various mediators that might explain the link between prosocial behaviors and well-being. For instance, studies have examined the role of basic psychological needs, social support, self-esteem, and positive affect as potential mediators [36,51,52]. To better understand how and when PB predicts WB, it is crucial to investigate the mediating mechanisms. Therefore, this study aims to examine the complicated conceptual model that investigates the mechanisms behind the link between PB and WB among vocational students, filling in these research gaps. Specifically, we will explore how basic psychological needs mediate the relationship between PB and WB.

### 2.2. The Mediating Effect of Basic Psychological Needs

The theory of basic psychological needs, developed by Deci and Ryan [53], posits that satisfying the needs for autonomy, competence, and relatedness is crucial for promoting individual growth and enhancing well-being. According to this theory, individuals have an innate tendency towards self-integration and growth, which relies on the fulfillment of these basic psychological needs. Autonomy need refers to the desire for self-determination and control over one’s life, competence refers to the desire for mastery and effectiveness, and relatedness refers to the desire for close connections with others. When these needs are satisfied, individuals are more likely to experience positive physical and mental health outcomes [54]. Conversely, when these needs are not met, individuals may become ill-adjusted and experience negative impacts on their well-being [55,56].

Research has consistently demonstrated that satisfying basic psychological needs positively influences well-being, leading to increased vitality, positive emotions, and self-esteem, and reduced anxiety and depression [57,58,59]. Moreover, it has been found that prosocial behavior plays a significant role in satisfying these basic psychological needs and contributing to overall well-being [60,61]. Prosocial behavior has been shown to fulfill individuals’ autonomy needs by allowing them to express their internal values and exercise a sense of control over their actions [36]. When individuals engage in acts of kindness or cooperation, they have the opportunity to make choices aligned with their personal values, thus satisfying their need for autonomy. Additionally, prosocial behavior fulfills competence needs by providing opportunities to demonstrate one’s skills and usefulness to others [60]. By engaging in prosocial behaviors, individuals can showcase their competencies and capabilities, contributing to their sense of competence and self-efficacy. Furthermore, prosocial behavior satisfies individuals’ relatedness needs by reducing social isolation and fostering social connections [53]. When individuals engage in prosocial acts, they often experience a sense of connection with others and strengthen their social bonds. By helping others or engaging in cooperative behaviors, individuals create opportunities for social interaction and belongingness, fulfilling their need for relatedness. These findings highlight the significance of prosocial behavior as a means of satisfying basic psychological needs and contributing to overall well-being.

Building upon the existing literature, we propose the following hypothesis:

**Hypothesis** **2.** *The satisfaction of basic psychological needs mediates the relationship between prosocial behavior and well-being among vocational students. Specifically, vocational students who engage in more prosocial behaviors will experience increased satisfaction with their basic psychological needs, which will be associated with higher levels of well-being.*

## 3. The Present Study

**Figure 1:**
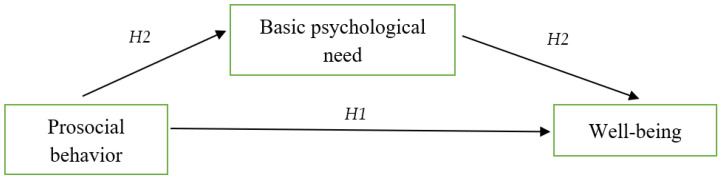
The proposed moderated mediation model.

This study aimed to investigate the relationship between prosocial behavior (PB) and well-being (WB) among vocational students and explore the underlying mechanisms involved. Specifically, two objectives were pursued. Firstly, the study examined the hypothesized positive association between PB and WB. Secondly, it tested the mediating role of basic psychological needs in the relationship between PB and WB. To analyze these relationships, a mediation model was proposed (see Figure 1).

### 3.1. Method

#### Participants

Convenience sampling was used to recruit participants from four vocational schools in Chongqing province, China. The sample size for this study was determined based on practical considerations and available resources, without explicit estimation of the minimum necessary sample size. A total of 267 vocational students volunteered to participate in the study. After excluding participants with incomplete responses and those who provided uniform answers to all questionnaire items, the final sample consisted of 221 participants. Among them, 89 (40.27%) were males, and 132 (59.72%) were females. The mean age of the participants was 20.16 years (*SD* = 1.57), ranging from 17 to 21 years. In terms of birthplace, 112 (50.68%) participants came from a village, 43 (19.46%) from a town, and 66 (29.86%) from a city. Moreover, 99 (44.80%) were only children, while 122 (55.20%) had siblings. A power analysis was conducted to determine the adequacy of the sample size for the mediation analysis, taking into account the hypothesized effect size, significance level, and statistical power. The results of the power analysis indicate that the sample size was sufficient to detect meaningful effects.

### 3.2. Measures

#### 3.2.1. Prosocial Behavior

Prosocial behavior was assessed using the “Interpersonal Attachment to Social Institutions Scale”, a self-report questionnaire developed by Gustavo Carlo [62], an American psychologist. The scale consists of six subscales, comprising a total of 23 items: altruistic (5 items), anonymous (5 items), public (4 items), compliant (2 items), emotional (4 items), and urgent (3 items). The scale was originally validated with a sample of American college students, demonstrating good reliability with alpha coefficients for the six subscales as follows: 0.74, 0.85, 0.78, 0.80, 0.75, and 0.63, respectively. In the present study, the revised questionnaire exhibited good reliability and validity, with an overall alpha coefficient of 0.862. The alpha coefficients for each subscale were as follows: public (0.739), anonymous (0.726), altruistic (0.752), compliant (0.679), emotional (0.777), and urgent (0.673). Confirmatory factor analysis (*CFA*) indicated good construct validity, with fit indices as follows: *χ2*/*df* = 4.19, GFI = 0.91, *CFI* = 0.93, *NFI* = 0.91, and *RMSEA* = 0.06.

#### 3.2.2. Basic Psychological Need

The Basic Psychological Needs Satisfaction Scale [53,63] was used to measure the extent to which the participants’ basic psychological needs were satisfied. The scale comprises 21 items, with 7 items assessing autonomy needs (e.g., “I feel free to decide how to live my life”), 6 items measuring competence needs (e.g., “I feel unable to perform well in many aspects of life”), and 8 items capturing relatedness needs (e.g., “I get along well with those around me”). Participants responded using a 5-point scoring system (1 = very inconsistent, 2 = somewhat inconsistent, 3 = neutral, 4 = somewhat consistent, 5 = very consistent). Scores for the three dimensions were computed after reverse scoring the appropriate items, with higher scores indicating greater satisfaction with basic psychological needs. The scale demonstrated good internal consistency in this study, with an overall reliability coefficient of 0.84. The reliability coefficients for each dimension were as follows: autonomy needs (0.77), competence needs (0.71), and relatedness needs (0.73). Confirmatory factor analysis yielded satisfactory fit indices: *χ2*/*df* = 2.508, *RMSEA* = 0.047, *CFI* = 0.903, and *TLI* = 0.923.

#### 3.2.3. Well-Being

In our study, we centered our investigation on the evaluation of a particular facet of well-being within the context of vocational students. To gauge well-being, we specifically assessed subjective well-being and vitality as key indicators. We derived a composite measure of overall well-being by summing the scores for subjective well-being and vitality [61,64]. Notably, our study deliberately excluded the incorporation of objective indicators of well-being, such as socioeconomic conditions. This deliberate exclusion aligns with our research’s targeted examination of this specific facet of well-being, emphasizing our commitment to this focused investigation.

Subjective well-being was measured using the Positive and Negative Affect Schedule (PANAS) [65]. The PANAS consists of 18 positive and negative emotion terms rated on a 5-point scale (1 = very mild or not at all, 2 = a little, 3 = moderately, 4 = to a considerable extent, 5 = very strongly). Higher scores indicate a greater intensity of emotional experience. In this study, the positive and negative emotion subscales demonstrated good internal consistency, with alpha coefficients of 0.92 and 0.83, respectively. Confirmatory factor analysis showed satisfactory fit indices: *χ2*/*df* = 2.902, *RMSEA* = 0.053, *CFI* = 0.962, and *TLI* = 0.953.

Life satisfaction was assessed using a 5-item scale in which the participants indicated their level of agreement with each statement on a 7-point scale (1 = strongly disagree, 2 = disagree, 3 = somewhat disagree, 4 = neither agree nor disagree, 5 = somewhat agree, 6 = agree, 7 = strongly agree). The scale demonstrated good internal consistency, with an alpha coefficient of 0.77. Confirmatory factor analysis yielded satisfactory fit indices: *χ2*/*df* = 1.510, *RMSEA* = 0.027, *CFI* = 0.998, and *TLI* = 0.993.

Vitality was assessed using the Chinese version of the Subjective Vitality Scale [66]. This scale employs a 7-point scale (1 = very nonconforming, 2 = relatively nonconforming, 3 = somewhat nonconforming, 4 = neutral, 5 = somewhat conforming, 6 = relatively conforming, 7 = very conforming), with higher scores indicating a higher level of subjective vitality. The scale demonstrated good internal consistency, with an alpha coefficient of 0.83. Confirmatory factor analysis yielded satisfactory fit indices: *χ2*/*df* = 1.972, *RMSEA* = 0.038, *CFI* = 0.996, and *TLI* = 0.988.

### 3.3. Procedure

The vocational school approved this study and adhered to the ethical standards outlined in the 1964 Helsinki Declaration and its later amendments. The participants were recruited to the survey anonymously by scanning a QR code in the online questionnaire. Before commencing the survey, the participants were informed about the voluntary nature of their participation and their ability to withdraw at any time during the study. An online informed consent form was presented to all participants, which they signed electronically. The online questionnaire took approximately 5 min to complete. Upon completion, the participants could enter a prize draw to win a bonus packet worth $1, $3, $5, or $10 as an incentive for participation. It is important to note that all procedures involving human participants in this study were conducted per the university’s approved guidelines, ethical standards, the 1964 Helsinki Declaration, and any subsequent amendments made to it.

### 3.4. Data Analysis

The data analysis process involved several steps. Firstly, a factor analysis was conducted to examine potential common method biases. Subsequently, confirmatory factor analysis was performed to assess the discriminant validity of the measurement scales. Descriptive statistics and Pearson correlations were then computed to explore the relationships among the study variables. To examine the mediating effect of basic psychological needs, we employed a simple mediation model using Model 4 of the PROCESS macro implemented in IBM SPSS Statistics (Version 25.0) and AMOS (Version 21.0), following the guidelines of Hayes [67]. This model allowed us to assess the direct, total, and indirect effects of prosocial behavior on well-being through basic psychological needs. The significance of mediation effects was determined using the bootstrap method with 5000 bootstrap samples and 95% confidence intervals [67]. Before conducting the mediation analysis, all continuous study variables, except for gender, birthplace, and only-child status, were standardized to facilitate interpretation. Additionally, control variables, including gender, age, and only-child status, were included in the analysis based on previous research [68,69] to account for potential confounding effects.

## 4. Results

### 4.1. Preliminary Analyses

To assess the presence of common method biases, Harman’s single-factor test was performed. The results revealed the extraction of eight factors with eigenvalues >1, accounting for 62.81% of the total variance. Notably, the first principal factor explained 30.08% of the variance, which did not meet the critical criterion of 40% [70]. These findings suggest the absence of standard method bias in the present study.

Discriminant validity was examined by comparing the square roots of the average variance extracted (*AVE*) with the correlation coefficients of the variables. The results indicate that all *AVE* values exceeded 0.5, and the square roots of *AVE* were greater than the correlation coefficients of any variables. This confirms the good discriminant validity of the measurement scales [71,72]. Moreover, the *HTMT* method (heterogeneity–monogenism ratio) was utilized to further verify the discriminant validity. The *HTMT* values presented in the table were used to assess the discriminant validity between the factors. Typically, an *HTMT* value below 0.85 (sometimes 0.9) indicates discriminant validity. In this study, all the *HTMT* values in the table fell within the standard range, demonstrating satisfactory discriminant validity. In summary, the data exhibited good discriminant validity, as indicated by the *AVE* values, correlation coefficients, and *HTMT* values. 

The means, standard deviations, and correlations among the main variables are presented in Table 1. The results indicate significant positive correlations among PB, basic psychological needs, and WB.

### 4.2. Testing the Mediating Effect

To examine Hypothesis 2, which proposed the mediating role of basic psychological needs between PB and WB, we employed Model 4 of the PROCESS macro, while controlling for gender, age, and only child [58]. The results presented in Table 2 reveal significant associations among the variables. Specifically, PB exhibited a positive relationship with basic psychological needs (*β* = 0.34, *t* = 28.83, *p* < 0.001, 95% *CI* = [0.30, 0.34]), and basic psychological needs were positively linked to WB (*β* = 0.25, *t* = 19.90, *p* < 0.001, 95% *CI* = [0.15, 0.19]). Moreover, when incorporating the mediator (basic psychological needs) into the model, PB remained positively associated with WB (*β* = 0.14, *t* = 10.85, *p* < 0.001, 95% *CI* = [0.18, 0.23]). These findings suggest that basic psychological needs partially mediate the relationship between PB and WB.

Further support for mediation was obtained through bootstrap analysis, which indicated a significant conditional indirect effect of PB on WB via basic psychological needs (indirect effect = 0.07, Boot *SE* = 0.01, Boot 95% *CI* = [0.07, 0.10]). The mediated effect accounted for 38.28% of the total effect.

Please refer to Table 2 for detailed results.

## 5. Discussion

The current study aimed to investigate the association between prosocial behavior (PB) and well-being (WB) among vocational students. A mediation model was constructed to examine whether basic psychological needs mediated the relationship between PB and WB. This study was the first to explore the relationship between PB and WB specifically among vocational students. The results indicate that PB was directly and indirectly related to WB through the mediator of basic psychological needs.

Consistent with our hypothesis, the findings of this study revealed a positive correlation between PB and WB among vocational students. This suggests that vocational students who engage in more PBs are more likely to experience higher levels of WB. By helping others, vocational students may feel capable, satisfied, and valued, perceiving these behaviors as aligned with their personal goals and contributing to better relationships. These factors can contribute to meeting their basic psychological needs, thereby increasing their WB.

Furthermore, the findings of this study align with previous empirical research that suggests PB can lead to positive psychological outcomes [73,74]. Thus, PB may serve as a stable predictor of psychological health. Additionally, this study extends prior research on altruistic behaviors [74,75] by demonstrating that altruistic behaviors can promote psychological health not only in older adults but also among vocational students.

### The Mediating Role of Basic Psychological Needs

As hypothesized, this study found that basic psychological needs partially mediated the relationship between PB and WB. Therefore, basic psychological needs served as an outcome of PB and a predictor of WB. In terms of the first path of the mediation process, the findings indicate a positive link between PB and basic psychological needs. This aligns with previous studies [76,77] that suggest that PB can trigger basic psychological needs. Vocational students who engage in more PBs are likely to derive successful experiences, pleasure, competence, autonomy, and self-satisfaction from their prosocial behaviors. The accumulation of these positive experiences can enhance their self-belief, positive self-schemata, and social performance in interactive situations [78], all of which contribute to higher levels of basic psychological needs. Bandura (1997) also emphasized the importance of previous experiences of success or failure as critical factors influencing behavior [79]. When individuals successfully help others, they may experience a sense of fulfillment or accomplishment. Therefore, the accumulation of successful experiences from PBs can improve vocational students’ satisfaction with basic psychological needs.

Regarding the second stage of the mediation link, this study demonstrated a positive relationship between basic psychological needs and WB, supporting previous research [80,81]. There are two possible explanations for this finding. First, basic psychological needs reflect individuals’ belief in their capacities [82]. Vocational students with higher levels of basic psychological needs are more likely to achieve their goals through their efforts, which increases their confidence, autonomy, and perceived control over their environment, ultimately contributing to higher levels of WB [83]. Second, individuals with high basic psychological needs tend to attribute success to their abilities and efforts, while attributing failure to external factors beyond their control. This attribution style can enhance their motivation levels. Moreover, this positive attribution style enables individuals to hold a positive attitude toward life and confidently cope with various life pressures. Therefore, the positive influence of basic psychological needs on vocational students’ WB is plausible.

In conclusion, this study contributes to the understanding of the relationship between PB and WB among vocational students. The findings support the positive association between PB and WB, highlighting the vocational students’ engagement in PBs and their positive impact on psychological well-being. The study also underscores the mediating role of basic psychological needs in this relationship, indicating that the fulfillment of basic psychological needs serves as a pathway through which PB promotes WB. These findings have theoretical and practical implications for promoting the well-being of vocational students by fostering PB and supporting basic psychological needs. Future research could explore additional factors that may influence the relationship between PB, basic psychological needs, and WB to further enhance our understanding of this complex interplay.

## 6. Implications

The study’s implications are twofold, encompassing both theoretical values and practical applications.

The theoretical implications of the study lie in confirming the mediating role of basic psychological needs in the relationship between PB and WB. This contribution deepens our understanding of the underlying mechanisms involved in this relationship and provides a framework for future research. By elucidating the importance of basic psychological needs satisfaction, researchers can refine existing theories and develop more comprehensive models that capture the complex interplay among PB, basic psychological needs, and WB.

Practically, this study suggests that interventions and programs should focus on promoting PB and supporting basic psychological needs. The findings indicate that encouraging vocational students to engage in PBs can have positive impacts on their mental health and overall well-being. Practitioners can play a crucial role in this process by emphasizing the positive aspects and benefits of PB, helping vocational students recognize the value of their altruistic behaviors. By fostering an understanding of the connection between PB and personal well-being, practitioners can enhance students’ motivation to engage in such activities and promote a sense of purpose and fulfillment.

In summary, while this study has limitations, it contributes to the existing literature by uncovering the mediating mechanisms in the relationship between PB and WB among vocational students. The implications of this study guide future research endeavors, suggesting the incorporation of objective measures, the exploration of moderating factors, and the consideration of context-specific basic psychological needs. These directions can further enhance our understanding of the relationship between PB and WB in diverse populations and cultural contexts. Additionally, the practical insights provided by the study inform interventions aimed at promoting the mental health and well-being of vocational students, highlighting the importance of supporting basic psychological needs and promoting PB as a means to enhance overall well-being.

### Limitations and Future Direction

The limitations identified in the study have important implications for future research. The use of self-report measures introduced subjective reporting bias, which highlights the need for incorporating objective measures or multiple data sources in future studies. By doing so, researchers can enhance the validity of the findings and provide a more robust understanding of the relationship between prosocial behavior (PB) and psychological well-being (WB). Additionally, exploring the moderating role of other interpersonal factors in this relationship would contribute to a more comprehensive understanding of the complex dynamics at play. By considering variables such as social support or empathy, researchers can uncover the contextual factors that influence the link between PB and WB.

Furthermore, the study suggests that using context-specific basic psychological needs would provide a more nuanced understanding of how PB relates to specific domains of psychological well-being. Different contexts, such as educational settings, work environments, or community engagement, may influence the extent to which basic psychological needs are satisfied and how they contribute to overall well-being. Therefore, future research should investigate the role of context in shaping the relationship between PB and WB, allowing for tailored interventions that address the unique needs of individuals in different domains.

The cross-sectional design of this study limits causal inference, as it only provides a snapshot of the relationships among variables at a particular point in time. To establish temporal relationships and identify the long-term effects of PB on WB, future longitudinal studies would be valuable. Longitudinal designs would allow researchers to track changes over time and explore how variations in PB influence subsequent changes in WB. By capturing the dynamic nature of these constructs, researchers can better understand the directionality and causality of the relationship. It is crucial to acknowledge that this study did not explicitly address the potential endogeneity problem, including the possibility of reverse causality between prosocial behavior and well-being. PB and WB can influence each other bidirectionally over time, raising concern of the endogeneity. Future research should consider more sophisticated analytical approaches, such as structural equation modeling, that account for such complexities and allow for a more rigorous examination of causal relationships.

Moreover, expanding the sample beyond Chinese vocational students would increase the generalizability of the findings to other populations and cultural contexts. Cultural factors can influence the interpretation and manifestation of PB and WB, and by including diverse samples, researchers can explore potential cultural variations in the relationships observed. This expansion would contribute to a more comprehensive understanding of the universal and culturally specific aspects of PB and WB, ultimately informing culturally sensitive interventions.

Lastly, it is important to acknowledge that the study employed a convenience sampling approach, which may introduce bias to the results. While this approach allowed for efficient data collection, the potential limitations associated with convenience sampling should be recognized. The findings of the study are based on a specific subset of participants who self-selected to participate, and therefore, the generalizability of the results to the broader population should be interpreted with caution. This limitation underscores the need for future research to consider diverse sampling methods to ensure a more representative sample and enhance the robustness of the findings.

## 7. Conclusions

The study found a positive association between PB and vocational students’ WB. PB was related to higher levels of WB, partially mediated by basic psychological needs. These findings contribute to the understanding of the role of PB in vocational students’ well-being and emphasize the importance to enhance the positive effects of PB. Practitioners and educators can use these findings to develop interventions promoting PB and improving vocational students’ well-being.

## Figures and Tables

**Table 1:** Descriptive statistics and intercorrelations between variables (*n* = 221).

Variables	*M ± SD*	1	2	3	4
1. Age	19.68 ± 1.57	1			
2. Prosocial behavior	3.56 ± 0.80	−0.05 **	1		
3. Basic psychological need	4.63 ± 0.76	−0.04 **	0.34 **	1	
4. Well-being	3.86 ± 0.64	−0.03 *	0.22 **	0.29 **	1

** *p* < 0.01, * *p* < 0.05.

**Table 2:** Testing the mediation effect of basic psychological need in the relation between PB and WB.

	Model 1 (DV:Well-Being)				Model 2 (DV:BPN)				Model 3 (DV:Well-Being)			
	*B*	*t*	*p*	*β*	*B*	*t*	*p*	*β*	*B*	*t*	*p*	*β*
Constants	3.42 **	65.39	0.00	-	3.5 **	57.11	0.00	-	2.70 **	43.33	0.00	-
Gender	−0.11 **	−6.74	0.00	−0.08	−0.01	−0.60	0.55	−0.01	−0.11 **	−6.80	0.00	−0.08
Age	−0.000	−1.215	0.224	−0.015	−0.001 *	−1.971	0.049	−0.023	−0.000	−0.76	0.45	−0.01
Only child	−0.02	−0.803	0.422	−0.010	0.010	0.484	0.629	0.006	−0.017	−0.95	0.34	−0.01
PB	0.17 **	18.14	0.00	0.22	0.32 **	28.83	0.00	0.34	0.11 **	10.85	0.00	0.14
BPN									0.21 **	19.90	0.00	0.25
*R* ^2^	0.06				0.12				0.11			
*F*	*F* (221) = 96.95, *p* = 0.00				*F* (221) = 211.13, *p* = 0.00				*F* (221) = 161.50, *p* = 0.00			

* *p* < 0.05, ** *p* < 0.01, IV = independent variable; DV = dependent variable; BPN = basic psychological need; PB = Prosocial behavior; WB = Well-being.

## Data Availability

The data presented in this study are available upon request from the corresponding author. The data are not publicly available due to privacy concerns.
